# Effect of calories-only vs physical activity calorie expenditure labeling on lunch calories purchased in worksite cafeterias

**DOI:** 10.1186/s12889-019-6433-x

**Published:** 2019-01-23

**Authors:** Anthony J. Viera, Ziya Gizlice, Laura Tuttle, Emily Olsson, Julie Gras-Najjar, Derek Hales, Laura Linnan, Feng-Chang Lin, Seth M. Noar, Alice Ammerman

**Affiliations:** 1 Department of Community and Family Medicine, Duke University School of Medicine, 2200 West Main Street, Suite 400, Durham, NC 27705 USA; 2 Center for Health Promotion and Disease Prevention, University of North Carolina at Chapel Hill, Chapel Hill, USA; 3 NC Translational and Clinical Sciences Institute, University of North Carolina at Chapel Hill, Chapel Hill, USA; 4 Sheps Center for Health Services Research, University of North Carolina at Chapel Hill, Chapel Hill, USA; 5 Department of Health Behavior, University of North Carolina at Chapel Hill Gillings School of Global Public Health, Chapel Hill, USA; 6 Department of Biostatistics, University of North Carolina at Chapel Hill Gillings School of Global Public Health, Chapel Hill, USA; 7 School of Media & Journalism, University of North Carolina at Chapel Hill, Chapel Hill, USA; 8 Department of Nutrition, University of North Carolina at Chapel Hill, Chapel Hill, USA

**Keywords:** Calorie labeling, Physical activity, Obesity prevention policy

## Abstract

**Background:**

Calorie labeling on restaurant menus is a public health strategy to guide consumer ordering behaviors, but effects on calories purchased have been minimal. Displaying labels communicating the physical activity required to burn calories may be a more effective approach, but real-world comparisons are needed.

**Methods:**

In a quasi-experimental study, we examined the effect of physical activity calorie expenditure (PACE) food labels compared to calorie-only labels on point-of-decision food purchasing in three worksite cafeterias in North Carolina. After a year of quarterly baseline data collection, one cafeteria prominently displayed PACE labels, and two cafeterias prominently displayed calorie-only labels. Calories from foods purchased in the cafeteria during lunch were assessed over 2 weeks every 3 months for 2 years by photographs of meals. We compared differences in purchased calorie estimates before and after the labeling intervention was introduced using longitudinal generalized linear mixed model regressions that included a random intercept for each participant.

**Results:**

In unadjusted models comparing average meal calories after vs before labeling, participants exposed to PACE labels purchased 40.4 fewer calories (*P* = 0.002), and participants exposed to calorie-only labels purchased 38.2 fewer calories (*P* = 0.0002). The small difference of 2 fewer calories purchased among participants exposed to PACE labeling vs calorie-only labeling was not significant (*P* = 0.90). Models adjusting for age, sex, race, occupation, numeracy level, and health literacy level did not change estimates appreciably.

**Conclusion:**

In this workplace cafeteria setting, PACE labeling was no more effective than calorie-only labeling in reducing lunchtime calories purchased.

## Background

Over one-third of adults in the United States (US) are obese, and obesity is a significant risk factor for heart disease, stroke, hypertension, diabetes, and certain cancers, in addition to all-cause mortality [1, 2]. The obesity epidemic has been linked to both dietary habits and inadequate levels of physical activity. Americans consume almost one-third of their daily calories from food purchased away from the home [3]. With higher calorie content and larger portion sizes, food purchased away from home has been implicated in the high prevalence of obesity [4, 5]. One policy approach to try to curb the obesity epidemic is the requirement included in the 2010 Patient Protection and Affordable Care Act requiring restaurants with 20 or more locations to post calorie information on their menu boards. However, evidence supporting this type of calorie labeling is mixed, with systematic reviews suggesting limited effectiveness in decreasing the amount of calories people order or consume [6, 7].

Calorie information alone may not be sufficient to motivate behavior change, especially when making a decision at the point of purchase (such as in a fast food or cafeteria line) where distractions and time-pressures are common. People may not fully appreciate the effect of caloric intake from individual food items on maintaining a healthy weight. Even if people understand the effect of eating too many calories, behavioral economic theory suggests that the lack of influence of calorie labeling may be due to its reliance on the “rational” system rather than the “intuitive” system. In other words, making use of calorie information may be hampered by limited time or willingness for cognitive processing. Framing calorie information by indicating the amount of physical activity required to burn calories may be more intuitive in that it translates to something (e.g., walking distance) people easily understand. Therefore, such re-framing may result in greater influence on point-of-purchase consumer behavior. An additional potential benefit of such labels is that they may promote physical activity.

In our pilot work, we found in a randomized, controlled trial using hypothetical fast-food restaurant scenarios that people selected a meal totaling fewer calories when shown our PACE labels vs calorie-only labels (826 cals vs 927 cals) [8]. To begin to explore whether such an effect is seen on actual behavior in real-world settings, we conducted a quasi-experimental trial to examine the effect of PACE labels compared to calorie-only labels on average purchased calories per meal during lunchtime in three worksite cafeterias.

## Methods

### Study overview

The overall design of the PACE Study has been previously published in detail [9]. In brief, we partnered with Blue Cross and Blue Shield of North Carolina (BCBSNC) to examine the effects of PACE labels compared to calorie-only labels in three worksite campus cafeterias. Employees who regularly ate lunch in the campus cafeterias were invited to participate in the study. During a pre-intervention year, we collected baseline data from participants in all three cafeterias. At the beginning of the second year of the study, one cafeteria had its food items prominently labeled with PACE labels and two cafeterias had their food items labeled with calorie-only information. The reasons for combining two cafeterias were (1) to maintain enough cohort participants over the duration of the study, and (2) to better balance the characteristics of employees.

### Primary outcome

The primary outcome was calories purchased during lunchtime as measured every 3 months over a 2-year period by individuals enrolled in two cohorts. This outcome was assessed at baseline (prior to labeling) and during the 1-year labeling intervention.

### Participant recruitment and eligibility

We advertised the study using paper and electronic flyers. Study coordinators actively recruited participants in the worksite cafeterias by setting up an informational table for employees to visit and to learn more about or sign up for the study. Enrollment of participants continued on a rolling basis throughout the baseline year to help compensate for attrition. To be eligible, a participant needed to (1) be a BCBSNC employee or contractor, and (2) eat lunch or be willing to eat lunch in the BCBSNC cafeteria at least 3 times per 5-day work week.

### Outcome measure

We collected detailed information on the lunch purchases made by the participants using a specially designed photo capture system. The system consisted of a touchscreen monitor and camera positioned above a shelf that allowed participants to take a picture of their entire meal contents. Photographs were saved with the participants’ study identification number, initials, date and site location. Study coordinators were on-site during all data collection periods to record details that may not have been evident from the photograph (e.g., dressings, condiments, soup or drink contents).

Participants submitted lunch photos over 2-week periods on a quarterly basis during the 24-month study period. Study staff analyzed all lunch photos and entered calorie information into a detailed database. For any items requiring estimation of portion sizes (e.g., self-serve items or self-built salads), Study coordinators used a food atlas along with their on-site notes to estimate portion sizes [9].

### Labeling intervention

Following the year of baseline data collection, one cafeteria received PACE labels which showed the calories in the food as well as an image of someone walking and the estimated number of miles needed to “burn off” the calories [9]. We determined PACE label values as previously described [9]. The two other cafeterias received calorie-only labels. The labels, which were bright green, bright blue, or bright yellow and measured 3 X 4 in., were prominently displayed above or beside each food item. For food prepared to order at the grill and deli, lists of commonly purchased items were posted with the PACE or calorie label [9]. For salad bars, we posted lists of common items as well as representative salads showing the sum of calories or PACE equivalent from all ingredients included. Beverage cooler doors were labeled with lists of all their beverages.

### Statistical considerations and analysis plan

We based sample size on the outcome of change in purchase calories before versus after the labeling intervention. For sample size calculations, we assumed standard deviation of purchased lunch meals is 350 cal and expected that the PACE labeling intervention would reduce purchase calories by 100 cal based on our preliminary study [8]. There were four measures of purchased calories (one for each quarter) prior to the intervention and four after the intervention. We analyzed purchased calorie data from all time points using a longitudinal generalized linear mixed model (GLMM) that included study groups, time, and study group by time interaction as fixed effects, each participant as random effect and a covariance structure that provided the best fit for the model to obtain estimates of slopes before and after introducing the calorie label interventions and compared between groups. We compared baseline sample characteristics of the two study groups using t-test and chi-square tests. Study groups differed significantly for age, sex, race, and occupation categories. These variables were included as fixed covariates along with literacy and numeracy scores (considered a priori) in a similar GLMM to compare slopes before and after the interventions between groups. In addition, a small number of participants changed worksite cafeterias prior to the intervention, so we analyzed participants using the same GLMMs based on the cafeteria in which they took > 50% of their lunch photos during the intervention year.

## Results

### Baseline characteristics of participants

**Fig. 1:**
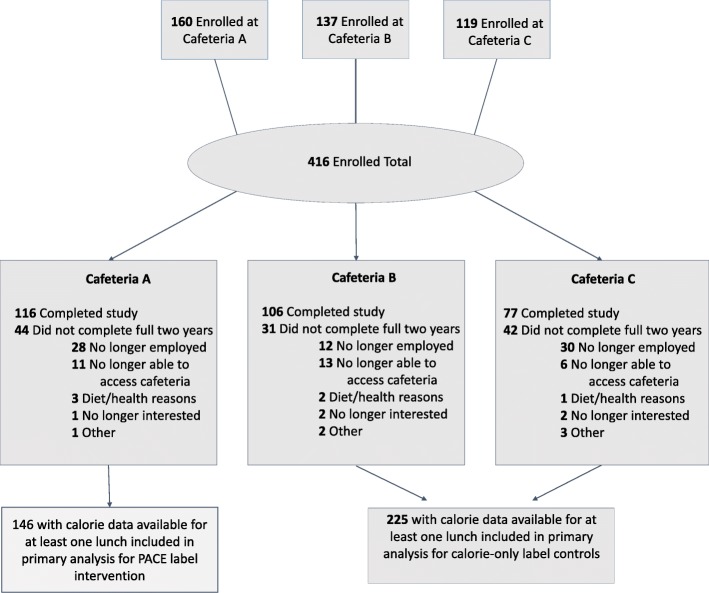
Enrollment and Retention

A total of 416 individuals initially consented to be in the study. Due to relocations, layoffs, and other life circumstances, some employees did not participate for the full 2 years. A total of 371 participants contributed lunchtime photographic lunch tray data to at least one of 8 time points (Fig 1). The majority of the 371 participants were female (78.4%), and the sample was racially diverse with 46% white and 44% Black (Table 1). The mean body mass index in the cohort at baseline was 32.0 kg/m^2^ and did not differ significantly between groups. The demographics of the cohort reflected those of the entire employee population. Characteristics that differed between the participants across worksites included age, sex, race and occupational roles.

**Table 1:** Characteristics of cohort participants

	Entire Cohort		PACE Labels		Calorie Only Labels	
	*N*	% or Mean(SD)	*N*	% or Mean(SD)	*N*	% or Mean(SD)
Age, Mean (SD)	371	42.2(10.1)	146	40.9(9.8)	225	43.0(10.3)
Female, %	291	78.4	106	72.6	185	82.2
Race, %						
White	171	46.1	60	41.1	111	49.3
Black or AA	164	44.2	65	44.5	99	44.0
Asian	36	9.7	21	14.4	15	6.7
Hispanic Ethnicity,%	18	4.9	10	6.9	8	3.6
Education Level,%						
High school	47	12.7	17	11.6	30	13.3
Technical or trade	31	8.4	9	6.2	22	9.8
Associate’s degree	55	14.8	19	13.0	36	16.0
Bachelor’s degree	141	38.0	65	44.5	76	33.8
Master’s or other advanced degree	97	26.2	36	24.7	61	27.1
Current Smoking,%						
Everyday	19	5.1	3	2.1	16	7.1
Some days	13	3.5	5	3.4	8	3.6
Adequate^a^ Numeracy Score, %	206	55.5	89	61.0	117	52.0
Total Literacy, Mean(SD)	371	4.8(1.5)	146	4.8(1.4)	225	4.8(1.5)
Self-reported health,%						
Excellent	63	17.0	26	17.8	37	16.4
Very Good	139	37.5	52	35.6	87	38.7
Good	142	38.3	61	41.8	81	36.0
Fair	25	6.7	6	4.1	19	8.4
Poor	2	0.5	1	0.7	1	0.4
Occupation category, %						
Administrative	67	18.1	16	11.0	51	22.8
Customer service or	84	22.7	36	24.7	48	21.4
Financial or technical	119	32.2	55	37.7	64	28.6
Environmental	1	0.3	0	0.0	1	0.5
Management	99	26.8	39	26.7	60	26.8
Marital status, %						
Single, never married	107	28.8	44	30.1	63	28.0
Married or domestic	200	53.9	77	52.7	123	54.7
Widowed	4	1.1	1	0.7	3	1.3
Divorced or separated	60	16.2	24	16.4	36	16.0
Total household income,%						
Less than $25,000	3	0.8	1	0.7	2	0.9
$25,000 - 49,999	111	29.9	39	26.7	72	32.0
$50,000 - 99,999	136	36.7	52	35.6	84	37.3
$100,000 or more	121	32.6	54	37.0	67	29.8
Weight (lbs.), Mean(SD)	278	195.4(51.4)	99	199.4(51.6)	179	193.2(51.1)
Body Mass Index, kg/m^2^	278	32.0(8.1)	99	32.6(7.9)	179	31.7(8.2)
BP Systolic (mmHg), Mean(SD)	278	117.5(13.3)	99	117.1(13.6)	179	117.6(13.1)
BP Diastolic (mmHg), Mean(SD)	278	74.6(10.1)	99	75.7(10.1)	179	73.9(10.0)
Total Cholesterol (mg/dL), Mean(SD)	278	188.7(39.6)	99	187.4(36.6)	179	189.4(41.2)
HDL Cholesterol(mg/dL), Mean(SD)	274	57.1(17.0)	98	56.1(15.6)	176	57.6(17.7)
LDL Cholesterol (mg/dL), Mean(SD)	255	108.0(33.1)	92	107.2(33.5)	163	108.4(32.8)
Triglycerides (mg/dL), Mean(SD)	273	122.7(86.8)	96	125.9(76.9)	177	120.9(91.6)
Fasting Blood Sugar (mg/dL), Mean(SD)	259	92.6(23.2)	93	90.9(20.4)	166	93.6(24.5)

^a^2 or 3 correct out of 3 items

### Average meal calorie purchases

During the baseline year, 371 of the enrolled participants submitted a total of 4721 lunch photographs. During the intervention year, 246 of the participants submitted 3237 photographs. The averages of meal calories over each data collection period in intervention vs. comparison groups are shown in Table 2. The averages were lower in the calories-only group at all eight time points.

**Table 2:** Estimates of Mean Meal Calories Purchased at Each Data Collection Time Point from Generalized Linear Mixed Models for Intervention (PACE Label) and Control (Calorie Only) groups

Time Point	Unadjusted Model Estimates		Adjusted Model Estimates^a^	
	PACE Labels (*n* = 146)	Calorie Only (*n* = 225)	PACE Labels (*n* = 146)	Calorie Only (*n* = 225)
1	628	581	622	583
2	611	581	605	585
3	622	604	617	607
4	579	577	574	580
Baseline	610	586	605	589
5	603	556	598	560
6	562	525	560	526
7	561	572	553	578
8	552	537	548	537
Intervention	570	546	565	550
Mean Change	40	38	40	39
Difference	2		1	

^a^Adjusted for age, sex, race, occupational role, and numeracy and literacy scores

In unadjusted models comparing average meal calories after vs before labeling, participants exposed to PACE labels purchased 40.4 fewer calories (*P* = 0.002), and participants exposed to calorie-only labels purchased 38.2 fewer calories (*P* = 0.0002). The small difference of 2 fewer calories purchased among participants exposed to PACE labeling vs calorie-only labeling was not significant (*P* = 0.90). Models adjusting for age, sex, race, occupational role, numeracy level, and health literacy level or those models that had the participants assigned based on > 50% of their lunch photos during the intervention year did not change estimates appreciably.

## Discussion

Previous research demonstrates that calorie information is unlikely by itself to motivate people to change their eating behaviors. Calorie information alone lacks context. That is, without additional information, people may not appreciate how calories, particularly of a single meal, fit into their overall daily intake and energy balance. It has been proposed that framing caloric information by indicating the amount of physical activity required to burn the calories may increase its influence on consumer behavior [9, 10]. The PACE Study was designed in part to examine whether a labeling strategy that conveys calorie information in such a format would lead to a change in calories purchased.

Few other studies have examined the effect of physical activity energy equivalent food labeling formats [7, 11]. This current study’s testing of PACE labeling was informed initially by qualitative work [12]. Then we conducted a hypothetical scenario study in which just over 800 respondents were randomized to be shown one of four menu label types: calories only, calories plus average minutes of walking, calories plus average miles of walking to burn the calories in the food item, or no additional information [8]. In a computer-based format, respondents shown the calories plus miles ordered an average of 194 fewer calories compared to no label and 101 fewer compared to those shown the calories-only label. It is interesting to note that in one US study, approximately 26% of participants reported they would like to see physical activity equivalents provided with calorie information [13].

In this current “real-world” study, however, cafeteria patrons exposed to PACE labels during their workday lunch did not purchase any fewer calories than those exposed to calorie-only labels.

One might question if employees of a health insurance company might have already been more fit or consumed a healthier diet than the general population, and thus it would be difficult to affect change through labeling. However, baseline data regarding BMI of the study sample suggests otherwise.

Prior to initiating the study, some of the cafeterias used limited calorie labeling on some of the foods, but the labels were quite small and seemingly largely ignored. In order to standardize the labels across study sites, we improved the size and prominence of the calorie only labels as well as testing the PACE labels which were similarly visible. It is possible that by enhancing the calorie-only labels, we increased their effectiveness [14]. It is possible that participants exposed to PACE labeling altered their behaviors in other ways that may have contributed to better health other than purchasing fewer lunch calories. They may have selected foods of improved nutritional value even if the calorie levels remained the same. In addition, it is possible that actual consumption was reduced in the PACE group, but we did not measure foods purchased but not eaten. Participants may have engaged in a greater amount of physical activity (which we will examine in a subsequent analysis). It is also possible that participants made alternative meal choices for subsequent meals (i.e., ate fewer calories for dinner at home), that we would not have captured. We do note that participants in both groups purchased fewer calories once labels were in place. However, we did not include a no-label group, so it is not clear whether this decline represents a labeling effect in general or a secular trend towards caloric reduction.

Strengths of our study include its cohort design, use of meal photographs, and a detailed food atlas to assess calorie information. As a cafeteria study, however, its findings may not generalize to other settings such as fast food restaurants. Another potential limitation is that we measured food purchased. It is possible that people ate less of their meals in one condition or another, but we did not measure actual food consumption.

## Conclusions

Overall, it appears that in the workplace cafeteria setting during lunchtime, PACE labeling is no more effective than calorie-only labeling in reducing calories of meals purchased. Additional analyses will examine effects of PACE labeling on physical activity. Future studies in alternative settings, especially fast food restaurants, also should be considered.

## Abbreviations

BCBSNC: Blue Cross Blue Shield of North Carolina
CHOW: Capturing healthy options at work
GLMM: Generalized linear mixed model
PACE: Physical activity calorie expenditure
